# Supplementary material for: Splice-Junction-Based Mapping of Alternative Isoforms in the Human Proteome
Source: Cell Rep. Author manuscript; Available in PMC 2020 Jan 15. (PMC6961840; doi:10.1016/j.celrep.2019.11.026)
Supplement: 2 [file NIHMS1546469-supplement-2.zip › DF1/PXD010154/Esophagus/Esophagus_27_PARN_ELSPAGSISKNSPATLFEVPDTSKENSNLHK.pdf]

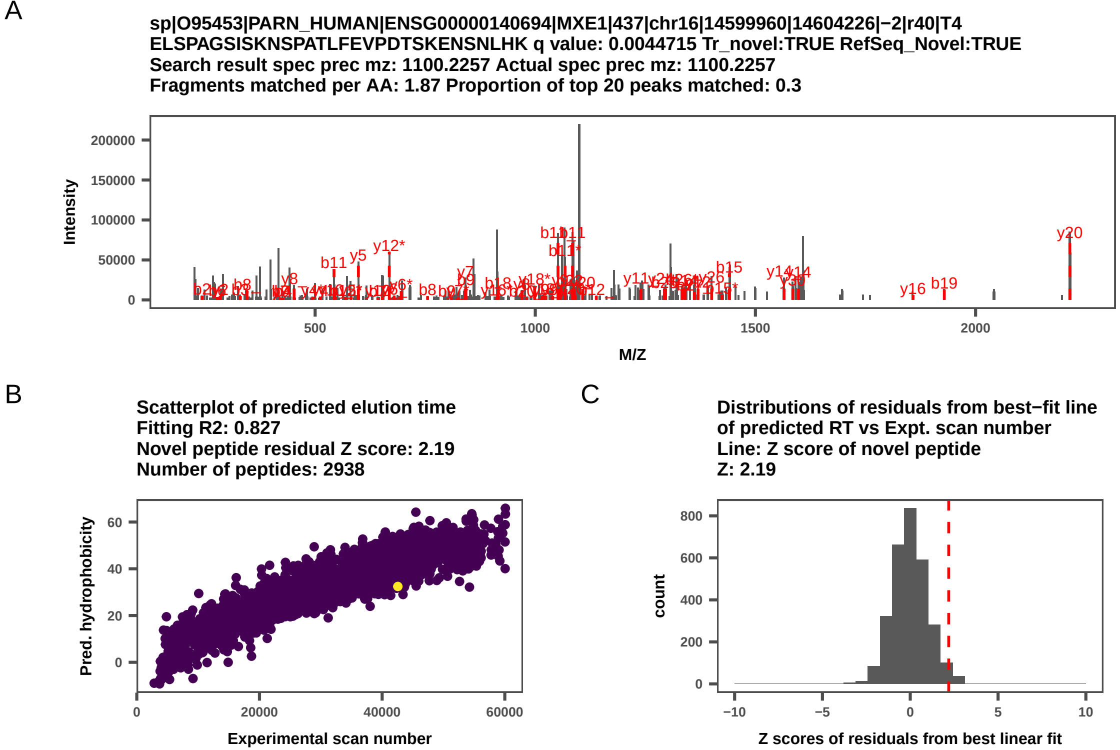
A
sp|O95453|PARN_HUMAN|ENSG00000140694|MXE1|437|chr16|14599960|14604226|−2|r40|T4
ELSPAGSISKNSPATLFEVPDTSKENSNLHK q value: 0.0044715 Tr_novel:TRUE RefSeq_Novel:TRUE
Search result spec prec mz: 1100.2257 Actual spec prec mz: 1100.2257
Fragments matched per AA: 1.87 Proportion of top 20 peaks matched: 0.3
Intensity
200000
150000
100000
50000
0
500
1000
1500
2000
M/Z
y20
b11
y12*
y5
b15
y16
b19
B
Scatterplot of predicted elution time
Fitting R2: 0.827
Novel peptide residual Z score: 2.19
Number of peptides: 2938
Pred. hydrophobicity
60
40
20
0
0
20000
40000
60000
Experimental scan number
C
Distributions of residuals from best−fit line
of predicted RT vs Expt. scan number
Line: Z score of novel peptide
Z: 2.19
count
800
600
400
200
0
−10
−5
0
5
10
Z scores of residuals from best linear fit
